# Changing awareness and sources of tobacco and e-cigarettes among children and adolescents in Great Britain

**DOI:** 10.1136/tc-2023-058011

**Published:** 2023-07-30

**Authors:** Jennie C Parnham, Charlotte Vrinten, Hazel Cheeseman, Laura Bunce, Nicholas S Hopkinson, Filippos T Filippidis, Anthony A Laverty

**Affiliations:** 1 Public Health Policy Evaluation Unit, Imperial College London, London, UK; 2 Action on Smoking and Health (ASH), London, UK; 3 NIHR Respiratory Biomedical Research Unit at the Royal Brompton & Harefield NHS Foundation Trust and Imperial College, London, UK

**Keywords:** Public policy, Advertising and Promotion, Packaging and Labelling

## Abstract

**Introduction:**

It is illegal in the UK to sell tobacco or nicotine e-cigarettes to people under the age of 18 years, as is displaying tobacco cigarettes at the point of sale. This paper examined changes in exposure to display of these products in shops and sources of these products among children and adolescent users over time

**Methods:**

Data from representative repeated online cross-sectional surveys of youth in Great Britain (11–18 years) were used (2018–2022; n=12 445). Outcome measures included noticing product displays and sources of e-cigarettes and tobacco cigarettes. Logistic regressions examined the associations of these outcome variables over time and with sociodemographic variables.

**Results:**

Of 12 040 participants with complete data, 10.1% used some form of nicotine product (4.2% cigarettes, 2.9% e-cigarettes, 3.0% both) at least occasionally. The likelihood of noticing tobacco cigarettes on display fell over time for both supermarkets (2018: 67.1% to 2022: 58.5%) and small shops (2018: 81.3% to 2022: 66.3%), but the likelihood of noticing e-cigarettes in supermarkets rose (2018: 57.4% to 2022: 66.5%). Sources of tobacco cigarettes did not differ over time, but e-cigarette users were more likely to get their e-cigarettes from small shops in 2022 (51.2%) vs 2019 (34.2%) (OR 2.02, 95% CI 1.24, 3.29).

**Conclusion:**

This study provides evidence that current policies to limit awareness of and access to both tobacco and e-cigarettes among adolescents in the UK may not be effective. UK policies on the advertising, promotion and sale of both tobacco and e-cigarettes need to be reinforced to deter use among children and adolescents.

**WHAT IS ALREADY KNOWN ON THIS TOPIC**

- The UK has a point-of-sale display ban for tobacco but does not have one for e-cigarettes. A recent rise in adolescents using e-cigarettes suggests minimum age restrictions for e-cigarettes may not be adequately enforced at present. Considering rapid changes in the tobacco market, it is important to examine the effectiveness of these policies for tobacco and e-cigarette use in adolescents.

**WHAT THIS STUDY ADDS**

- This study examines the awareness of nicotine products on display among adolescents and the sources of nicotine products among adolescent users. We found that noticing tobacco cigarettes in all shops fell over time, whereas noticing e-cigarettes in supermarkets rose over time. There was little evidence that sources of tobacco cigarettes changed, but adolescent users were more likely to source their e-cigarettes from small shops in 2022 compared with 2019.

**HOW THIS STUDY MIGHT AFFECT RESEARCH, PRACTICE OR POLICY**

- The study highlights the differences between levels of exposure to tobacco and e-cigarettes. Children and adolescents are also still able to obtain both tobacco and e-cigarettes from shops despite existing regulations prohibiting sale to minors. UK policies on the advertising, promotion and sale of both tobacco and e-cigarettes need to be reinforced to deter use among children and adolescents.

## Introduction

 Advertising, promotion and sponsorship of tobacco products are known to be key methods of encouraging tobacco use.^1^ Children and adolescents are particular targets due to the need for the tobacco industry to recruit tobacco users.^2^ The retail environment is a key location for such advertising and promotion, with evidence linking exposure to increased smoking desire and cigarette purchasing.^3^,^5^ WHO advocates a complete ban on the display of tobacco at the point of sale,^6^ although only 22% of the global population live in countries with total tobacco advertising bans.^7^ Although there are less data on the issue of advertising and promotion of e-cigarette due to their recent emergence, WHO invites countries to ‘consider prohibiting or regulating ENDS/ENNDS, including as tobacco products, medicinal products, consumer products, or other categories, as appropriate’.^8^

Another key strategy to reduce uptake and use of tobacco among young people is reducing access through enforcing a minimum age of sale of at least 18 years.^9^ Increased minimum age of sale policies are designed to reduce direct access to tobacco products as well as access through friends and social sources.^10^ These policies are known to be effective: for example, increasing the legal age of sale from 16 to 18 years in England was linked to both an immediate and long-term fall in prevalence of tobacco smoking among 16 and 17 year-olds.^11 12^ For e-cigarettes, WHO also recommends minimum ages of sale for e-cigarettes.^7^ The latest WHO report on the global tobacco epidemic found that 69 countries (40%) had minimum age of sale legislation for e-cigarettes, with 40 of these countries being in the European Region. This is compared with 90% of countries with age restriction for policies for tobacco.

The UK has legislation in place to restrict promotion, advertising and sponsorship as well as access to tobacco products among children under the age of 18 years.^13^ Tobacco displays at the point of sale were banned in two stages: in large shops (with >280 m^2^ floor area) in April 2012 and then in all shops from April 2015.^14^ This legislation reduced exposure to tobacco advertising among children in both England and Scotland, although exposure levels have remained high.^15 16^ Regulations for the advertising of e-cigarettes are less stringent, although this is not allowed on the television, radio, online or in print media.^17^ There is no equivalent point-of-sale display ban for e-cigarettes in the UK. Selling tobacco to people under the age of 18 is not allowed, nor is sale of nicotine-containing e-cigarettes. Despite these regulations, e-cigarette use is reported by approximately 9% of 11–18 year-olds (including occasional and regular use), with use more common among those reporting seeing e-cigarette promotion.^18 19^ This paper examined changes in exposure to display of tobacco and e-cigarettes among children and adolescents in Great Britain from 2018 to 2022, and examined the sources of tobacco and e-cigarettes among product users.

## Methods

### Data source

Data were taken from an annual cross-sectional survey of adolescents (11–18 years), the Action on Smoking and Health Smokefree Great Britain Youth Survey, 2018–2022. This online survey has been run by YouGov since 2013, which invites a random sample of parents of 11–18 year-olds from their membership panel to participate by email each year. Informed consent from both the parent and the child was given before the adolescent completed the questionnaire. The adolescent survey includes questions on their sociodemographic characteristics, their awareness of and attitudes towards tobacco and e-cigarette product use, as well as their own use of these products. A total of 12 445 participants responded, of which 405 were excluded due to missing data (social grade n=164; product use n=241). Weights, supplied by the survey company, were used to ensure the responses were representative of all adolescents in Great Britain aged 11–18 years.

### Product use

All participants were asked whether they used cigarettes and e-cigarettes, respectively. Participants were coded as ‘*Current cigarette users*’ if they responded ‘sometimes but less than one a week’, ‘between one and six cigarettes a week’ or ‘more than six cigarettes a week’. Participants were coded as ‘*Former cigarette users’* if they responded ‘used in the past but not now’ or ‘tried once or twice’. Similarly, participants were coded as ‘*Current e-cigarette users*’ if they responded they use e-cigarettes ‘sometimes but no more than once a month’, ‘more than once a month, but less than once a week’, ‘more than once a week but not daily’ and ‘every day’. Participants were coded as ‘*Former e-cigarette users’* if they responded ‘used in the past but not now’ or ‘tried once or twice’. For both products, ‘*Non-users’* were participants who responded that they had never used the product. A nicotine product use summary variable combining answers to both e-cigarette use and tobacco smoking was also created. Participants were defined as a ‘*non-user’* if they did not currently use tobacco cigarettes and e-cigarettes, an *‘E-cigarette user only’* if they reported currently using e-cigarettes but not tobacco cigarettes, a ‘*Tobacco cigarette user only’* if they reported currently using tobacco cigarettes but not e-cigarettes and a ‘*Dual user’* if they reported currently using both.

### Display of products

Participants who responded they were aware of e-cigarettes (n=11 188) were asked ‘When you go into supermarkets, how often, if at all do you notice e-cigarettes on display?’ with a similar question for small shops (defined in the question as ‘corner shops/newsagents/off-licences’, which would all be considered as convenience stores in the USA). The same two questions were asked about tobacco cigarettes in supermarkets and small shops. The six possible responses were recoded into a binary variable (‘*Notice’*=‘Every time’, ‘Most times’, ‘Sometimes’ and ‘Hardly ever’; *‘Do not notice’*=‘Never’ and ‘I never go to supermarkets/small stores’).

### Sources of nicotine products

Participants who currently used tobacco cigarettes or e-cigarettes were asked where they usually source these, with separate questions for e-cigarette (n=717) and tobacco cigarette users (n=867). The participants could choose multiple sources from 16 categorical options and were given an option of an open-ended ‘other’ response. All of the responses were coded into four binary variables which were not mutually exclusive: ‘*Bought: supermarket*’, ‘*Bought: small shop*’ (such as newsagents, garages and vape shops), ‘*Bought: online’* and ‘*Acquired other’*. ‘*Acquired other’* represents a range of sources, including acquiring from a friend or family member, or purchase from a street market. Only four individuals responded that they bought tobacco cigarettes online, therefore this was not used as a separate category in the analysis. See online supplemental table 1 for a full list of responses and categorisation.

### Covariates

Covariates considered in the analysis included survey year (2018–2022), age group (11–13, 14–15, 16–17 and 18 years old), gender (male or female), social grade of the household (based on National Readership Survey classification of occupations^20^ and classified as ABC1 (higher) vs C2DE (lower)), country (England, Scotland or Wales), current e-cigarette use by others in the household (yes or no) and current tobacco smoking by others in the household (yes or no).

### Statistical analysis

Data collection on noticing display of tobacco and e-cigarette products began in 2018, while the question on sources of e-cigarettes was added in 2019. Weighted χ^2^ tests were used to determine difference in covariates and nicotine product use across survey years.

To determine factors associated with noticing nicotine products on display in supermarkets and small shops, we ran logistic regression models separately for both e-cigarettes and tobacco cigarettes. Covariates included all of those listed above.

Logistic regression models were also used to examine factors associated with sources of nicotine products among current users. Separate models were run for e-cigarette and tobacco cigarette outcomes. We tested interactions between age and survey year (both as continuous variables) for each model.

Statistical analyses were weighted to ensure the findings were representative to adolescents aged 11–18 years in Great Britain.

### Sensitivity analysis

To test the results were robust to possible misclassification bias, the variables for noticing e-cigarettes and tobacco cigarettes in supermarkets and small shops were recoded. We repeated the logistic regression models with *‘Do not notice’* recategorised to include ‘Hardly ever’, ‘Never’ and ‘I never go to supermarkets/small stores’.

## Results

The analytical sample contained 12 040 participants of which 10 453 had complete data on questions related to noticing nicotine products on display, 608 were current e-cigarette users with complete data on questions relating to source of e-cigarettes (years 2019–2022) and 831 were current tobacco cigarette users with complete data on questions relating to sources of tobacco cigarettes (years 2018–2022) (see online supplemental figure 1 for full details of exclusions).

In the full sample, 51% of participants were female, 71% were in higher (ABC1) social grades and 86% lived in England (table 1). Across survey years, there were significant differences in the age distribution of participants, with a lower proportion of participants aged 18 years in 2021 compared with other years. A greater proportion of participants were current e-cigarette (5.9%) or dual users (4.3%) in 2022 compared with 2018 (both 1.9% in 2018). The proportion of participants living in a household where e-cigarettes were used was higher in 2022 (21.3%) compared with 2018 (17.4%), whereas for smoking these proportions were similar (8.5% in 2022 compared with 9.0% in 2018).

**Table 1:** Characteristics of 12 040 UK adolescents who participated in the ASH Smokefree Great Britain Survey 2018–2022

Characteristic	2018(n=2222)	2019(n=2466)	2020(n=2461)	2021(n=2482)	2022(n=2409)	Total(N=12 040)
Age (years), n (%)						*
11–13	684 (30.8)	680 (27.6)	703 (28.6)	757 (30.5)	705 (29.3)	3529 (29.3)
14–15	561 (25.2)	546 (22.1)	569 (23.1)	577 (23.2)	615 (25.5)	2868 (23.8)
16–17	506 (22.8)	718 (29.1)	723 (29.4)	750 (30.2)	658 (27.3)	3355 (27.9)
18	471 (21.2)	522 (21.2)	466 (18.9)	398 (16.0)	431 (17.9)	2288 (19.0)
Gender, n (%)						
Male	1095 (49.3)	1192 (48.3)	1231 (50.0)	1191 (48.0)	1204 (50.0)	5913 (49.1)
Female	1127 (50.7)	1274 (51.7)	1230 (50.0)	1291 (52.0)	1205 (50.0)	6127 (50.9)
Social grade, n (%)						
ABC1 (higher)	1520 (68.4)	1781 (72.2)	1736 (70.5)	1754 (70.7)	1703 (70.7)	8494 (70.5)
C2DE (lower)	702 (31.6)	685 (27.8)	725 (29.5)	728 (29.3)	706 (29.3)	3546 (29.5)
Country, n (%)						
England	1954 (87.9)	2120 (86.0)	2129 (86.5)	2123 (85.5)	2085 (86.6)	10 411 (86.5)
Wales	100 (4.5)	124 (5.0)	116 (4.7)	115 (4.6)	105 (4.4)	560 (4.7)
Scotland	168 (7.6)	222 (9.0)	216 (8.8)	244 (9.8)	219 (9.1)	1069 (8.9)
Current nicotine product use status†, n (%)						*
Dual user	43 (1.9)	80 (3.2)	81 (3.3)	55 (2.2)	104 (4.3)	363 (3.0)
E-cigarette user	42 (1.9)	58 (2.4)	54 (2.2)	59 (2.4)	141 (5.9)	354 (2.9)
Cigarette user	117 (5.3)	128 (5.2)	124 (5.0)	70 (2.8)	65 (2.7)	504 (4.2)
Non-user	2020 (90.9)	2200 (89.2)	2202 (89.5)	2298 (92.6)	2099 (87.1)	10 819 (89.9)
Current e-cigarette use in the household, n (%)						*
Yes	354 (17.4)	366 (16.0)	387 (17.1)	352 (16.1)	457 (21.3)	1,916 (17.6)
(Missing)	190	185	200	299	267	1,141
Current tobacco smoking in the household, n (%)						
Yes	194 (9.0)	184 (7.7)	183 (7.6)	187 (7.7)	199 (8.5)	947 (8.1)
(Missing)	68	63	46	68	63	308

* P<0.05 in Pearson’s χ2 Chi-squaredtest for the difference in covariates across survey years.

† ‘*Non-user’*=not using tobacco cigarettes or e-cigarettes, *‘E-cigarette user only’*=using e-cigarettes but not tobacco cigarettes, ‘*Tobacco cigarette user only’*=using tobacco cigarettes but not e-cigarettes, ‘*Dual user’*=using tobacco cigarettes and e-cigarettes. Cigarette users included responses of ‘sometimes but less than one a week’ to ‘more than six cigarettes a week’. E-cigarette users included ‘sometimes but no more than once a month’ to ‘every day’.

ASHAction on Smoking and Health

### Factors associated with noticing nicotine products on display in supermarkets and small stores

The proportion of the sample that noticed tobacco cigarettes on sale in supermarkets fell from 67% in 2018 to 59% in 2022 (table 2**,** model 1). Corresponding figures for noticing tobacco at least sometimes were 33.0–27.4%. In the adjusted logistic regression, this corresponded to a 29% (OR 0.71; 95% CI 0.61, 0.82) lower odds of noticing tobacco cigarettes on display in supermarkets in 2022 than 2018. Noticing cigarettes in small shops also fell over time (2018: 81%; 2022: 66%). There was a lower likelihood of noticing cigarettes in small shops for 2019–2022 compared with 2018.

**Table 2:** Adjusted logistic regression showing the likelihood of noticing e-cigarettes and tobacco cigarettes on sale in supermarkets and small shops (n=10 453)

	Model 1: tobacco cigarettes				Model 2: e-cigarettes			
	Supermarket		Small shop		Supermarket		Small shop	
Characteristic	n(%)	aOR(95% CI)	n(%)	aOR(95% CI)	n(%)	aOR(95% CI)	n(%)	aOR(95% CI)
Total sample	6947 (64.1)		7701 (71.7)		7183 (66.5)		7668 (71.52)	
Survey year								
2018	1198 (67.1)	Ref	1444 (81.3)	Ref	1040 (57.4)	Ref	1269 (70.8)	Ref
2019	1566 (67.0)	1.01 (0.87, 1.17)	1667 (72.8)	0.61***(0.51, 0.72)	1602 (69.4)	1.78*** (1.54, 2.06)	1631 (71.1)	1.03 (0.89, 1.20)
2020	1531 (66.2)	0.99 (0.85, 1.14)	1665 (72.8)	0.62***(0.52, 0.73)	1667 (73.0)	2.16*** (1.86, 2.50)	1711 (75.3)	1.30*** (1.12, 1.52)
2021	1364 (61.9)	0.80** (0.69, 0.93)	1477 (67.0)	0.46***(0.39, 0.54)	1426 (64.5)	1.44*** (1.24, 1.66)	1506 (68.6)	0.93 (0.80, 1.09)
2022	1288 (58.5)	0.71*** (0.61, 0.82)	1448 (66.3)	0.45***(0.38, 0.54)	1448 (66.5)	1.57*** (1.35, 1.81)	1551 (71.6)	1.07 (0.92, 1.26)
Gender								
Male	3286 (62.6)	Ref	3696 (70.8)	Ref	3392 (64.6)	Ref	3693 (70.7)	Ref
Female	3661 (65.6)	1.17*** (1.06, 1.28)	4005 (72.7)	1.12*(1.02, 1.23)	3791 (68.6)	1.22*** (1.12, 1.34)	3975 (72.4)	1.10* (1.00, 1.21)
Age								
18	1602 (81.0)	Ref	1672 (84.7)	Ref	1577 (79.8)	Ref	1635 (82.8)	Ref
16–17	2319 (81.0)	0.99 (0.85, 1.15)	2434 (85.0)	1.06 (0.90, 1.25)	2303 (78.9)	1.00 (0.86, 1.17)	2373 (82.4)	1.02 (0.87, 1.20)
14–15	1465 (57.2)	0.33*** (0.29, 0.38)	1740 (67.6)	0.41*** (0.35, 0.48)	1604 (62.3)	0.45*** (0.39, 0.52)	1800 (69.8)	0.52*** (0.45, 0.61)
11–13	1561 (52.4)	0.27*** (0.24, 0.31)	1855 (61.6)	0.32*** (0.27, 0.37)	1699 (56.9)	0.36*** (0.32, 0.42)	1860 (61.8)	0.37*** (0.32, 0.43)
Social grade								
ABC1 (higher)	5027 (65.5)	Ref	5529 (72.7)	Ref	5141 (67.2)	Ref	5468 (71.9)	Ref
C2DE (lower)	1920 (60.6)	0.83*** (0.75, 0.91)	2172 (69.4)	0.87**(0.78, 0.96)	2042 (65.0)	0.90 (0.82, 1.00)	2200 (70.6)	0.93 (0.84, 1.04)
Country								
England	6074 (64.9)	Ref	6719 (72.5)	Ref	6242 (67.0)	Ref	6657 (72.0)	Ref
Wales	313 (62.0)	0.92 (0.75, 1.14)	355 (71.2)	0.97 (0.77, 1.21)	338 (66.7)	1.00 (0.81, 1.25)	369 (73.4)	1.09 (0.87, 1.38)
Scotland	560 (57.0)	0.69*** (0.60, 0.81)	627 (64.3)	0.65*** (0.56, 0.77)	603 (61.6)	0.77*** (0.66, 0.89)	642 (65.6)	0.71*** (0.61, 0.84)
Current nicotine product use status								
Non-user	6092 (62.7)	Ref	6757 (70.2)	Ref	6236 (64.6)	Ref	6690 (69.7)	Ref
E-cigarette only	267 (79.7)	1.62** (1.19, 2.21)	295 (88.2)	2.25*** (1.55, 3.27)	298 (88.8)	2.64*** (1.81, 3.84)	307 (91.4)	2.84*** (1.88, 4.29)
Tobacco cigarette only	326 (78.2)	1.17 (0.88, 1.55)	362 (87.9)	1.76*** (1.28, 2.43)	351 (83.4)	1.82*** (1.35, 2.46)	365 (87.6)	2.00*** (1.44, 2.79)
Dual user	262 (78.4)	1.28 (0.94, 1.75)	287 (85.5)	1.56* (1.10, 2.21)	298 (89.2)	2.36*** (1.61, 3.46)	306 (91.4)	2.56*** (1.69, 3.89)
E-cigarette use in the household								
No	5684 (63.6)	Ref	6281 (71.0)	Ref	5725 (64.2)	Ref	6150 (69.6)	Ref
Yes	1263 (66.4)	1.07 (0.94, 1.22)	1420 (75.2)	1.16*(1.01, 1.33)	1458 (77.4)	1.74*** (1.52, 2.00)	1518 (80.5)	1.63*** (1.41, 1.88)
Tobacco smoking in the household								
No	6322 (63.3)	Ref	7029 (71.1)	Ref	6515 (65.6)	Ref	6981 (70.7)	Ref
Yes	625 (73.4)	1.58*** (1.31, 1.91)	672 (78.8)	1.36**(1.11, 1.67)	668 (77.9)	1.54*** (1.26, 1.88)	687 (81.0)	1.42*** (1.15, 1.74)

N (%)=number and per cent of participants who noticed e-cigarettes or tobacco cigarettes on display within each stratum of the sociodemographic variables.

*P<0.05, **p<0.01, ***p<0.001.

aORadjusted OR

Noticing tobacco cigarettes on display in supermarkets and small shops was significantly less likely for those who were younger (11–13 and 14–15 vs 18 years), those in lower compared with higher social grades and Scottish compared with English participants. Those who lived in a household with current tobacco cigarette use were significantly more likely to notice tobacco cigarettes in supermarkets and small shops. All forms of nicotine product use (e-cigarette only, tobacco cigarette only and dual use) were associated with a significantly higher likelihood of noticing tobacco cigarettes in small shops compared with non-users. In supermarkets, only being an e-cigarette user was associated with a higher likelihood than non-users.

The proportion of the sample that noticed e-cigarettes in supermarkets rose from 57.4% in 2018 to 66.5% in 2022 (table 2, model 2). Corresponding figures for noticing e-cigarettes at least sometimes were 23.7–36.4%. In the adjusted logistic regression, there was a higher odds of noticing e-cigarettes in supermarkets for all years (2019–2022) than in 2018. Participants were more likely to notice tobacco cigarettes in small shops than supermarkets at baseline (81.3% vs 67.1%), with the same being true for e-cigarettes. The proportion of participants noticing e-cigarettes in small shops was higher at baseline than for supermarkets (2018 e-cigarettes: 70.8% vs 2018 tobacco cigarettes: 57.4%). Overall, there was not strong evidence that the likelihood of noticing e-cigarettes in small shops changed over time.

The likelihood of noticing e-cigarettes on sale in both supermarkets and small shops was statistically significantly lower for younger compared with older participants (11–13 and 14–15 vs 18 years) and those in Scotland compared with England. The likelihood was significantly higher for females compared with males, those who used nicotine products (e-cigarette only, tobacco cigarette only and dual use) compared with non-users and for participants who lived in households where people used e-cigarettes or smoked tobacco cigarettes compared with those who did not.

### Sources of tobacco cigarettes among current tobacco cigarette users

Overall, 25.1% of participants who smoked cigarettes sourced them from supermarkets, 50.6% sourced them from small shops and 58.1% acquired them from other sources (table 3). In adjusted logistic regression, there was no evidence that sources of tobacco cigarettes changed over time. Compared with 18-year-old users, younger cigarette users (16–17 and 14–15 years) were less likely to buy their cigarettes from supermarkets or small shops but were roughly three times more likely to acquire tobacco cigarettes from other sources than those aged 18 years (OR 3.51; 95% CI 2.11, 5.85 and OR 2.81; 95% CI 2.02, 3,91, respectively). Participants living in a household with someone smoking tobacco cigarettes were more likely to buy their tobacco cigarettes from a supermarket (OR 1.61; 95% CI 1.01, 2.59) or small shops (OR 2.26; 95% CI 1.46, 3.50).

**Table 3:** Fully adjusted and weighted logistic regression for likelihood of acquiring tobacco cigarettes from different sources in UK adolescents (n=831)

	Supermarket		Small shop		Acquired other	
Characteristic	n (%)	aOR (95% CI)	n (%)	aOR (95% CI)	n (%)	aOR (95% CI)
Total sample	221 (25.1)		422 (50.1)		474 (58.1)	
Survey year						
2018	41 (26.1)	Ref	77 (49.5)	Ref	88 (58.4)	Ref
2019	59 (26.7)	1.09 (0.64, 1.85)	91 (45.0)	0.89 (0.56, 1.40)	120 (62.7)	1.12 (0.70, 1.78)
2020	54 (26.1)	1.13 (0.66, 1.91)	94 (47.0)	0.98 (0.62, 1.56)	119 (61.0)	0.95 (0.60, 1.51)
2021	40 (32.0)	1.40 (0.78, 2.50)	68 (56.3)	1.44 (0.87, 2.39)	67 (52.4)	0.67 (0.40, 1.14)
2022	27 (15.6)	0.56 (0.30, 1.04)	92 (56.1)	1.34 (0.81, 2.22)	80 (53.3)	0.71 (0.42, 1.19)
Gender						
Male	109 (25.4)	Ref	213 (51.7)	Ref	226 (56.1)	Ref
Female	112 (24.8)	0.98 (0.70, 1.38)	209 (48.2)	0.87 (0.65, 1.17)	248 (60.3)	1.13 (0.83, 1.53)
Age						
18	137 (40.8)	Ref	193 (57.3)	Ref	149 (44.7)	Ref
16–17	54 (15.5)	0.26*** (0.17, 0.38)	156 (44.7)	0.56** (0.41, 0.78)	228 (68.5)	2.81*** (2.02, 3.91)
14–15	14 (15.6)	0.24*** (0.13, 0.45)	41 (43.5)	0.49** (0.31, 0.78)	71 (71.6)	3.51*** (2.11, 5.85)
11–13	16 (27.3)	0.47* (0.25, 0.90)	32 (56.8)	0.89 (0.48, 1.63)	26 (42.2)	0.94 (0.51, 1.70)
Social grade						
Middle class	164 (25.6)	Ref	287 (47.3)	Ref	352 (60.0)	Ref
Working class	57 (24.0)	0.92 (0.62, 1.35)	135 (57.4)	1.53* (1.09, 2.14)	122 (53.1)	0.71* (0.50, 0.99)
Country						
England	189 (24.9)	Ref	360 (50.3)	Ref	405 (58.8)	Ref
Wales	13 (40.0)	1.97 (0.89, 4.36)	18 (52.7)	0.87 (0.44, 1.74)	18 (45.2)	0.63 (0.32, 1.22)
Scotland	19 (20.3)	0.89 (0.48, 1.64)	44 (46.5)	0.87 (0.54, 1.40)	51 (58.9)	0.94 (0.58, 1.52)
Tobacco cigarette use in the household						
No	178 (24.1)	Ref	329 (46.4)	Ref	400 (59.4)	Ref
Yes	43 (29.8)	1.61* (1.01, 2.58)	93 (65.7)	2.26*** (1.46, 3.50)	74 (52.7)	0.77 (0.50, 1.18)

N (%)=number and per cent of tobacco cigarette users who obtained cigarettes through this source within each stratum of the sociodemographic variables. Tobacco cigarette sources are not mutually exclusive, therefore percentages do not add to 100% across sources.

*P<0.05, **p<0.01, ***p<0.001.

aORadjusted OR

**Figure 1:**
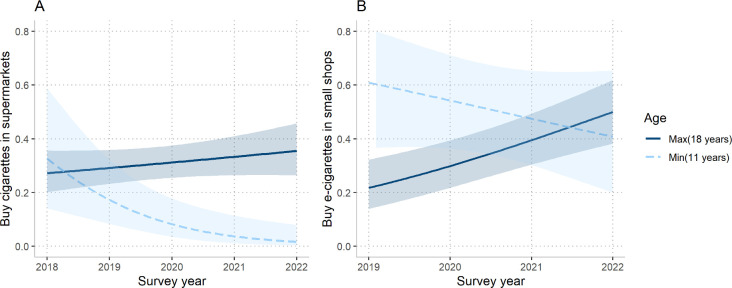
Interaction between age and survey year in logistic regression exploring sources of nicotine products in UK adolescents. (A) Interaction between age and survey year for the likelihood of buying tobacco cigarettes from supermarkets (n=831). (B) Interaction between age and survey year for the likelihood of buying e-cigarettes from small shops (n=608). Only the predicted likelihoods for the minimum (11 years) and maximum (18 years) ages are shown for clarity.

Interactions between age and survey year were tested for all regression models and were statistically significant for buying tobacco cigarettes from supermarkets, showing that for each year younger participants were less likely to buy cigarettes from supermarkets over time (figure 1A). For example, among 11 year-olds, there was a significant decreasing trend in buying cigarettes from supermarkets from over 30% in 2018 to less than 10% in 2022. The likelihood had a slight increase for 18 year-olds, but the CIs between 2018 and 2022 overlapped.

### Sources of e-cigarettes among current e-cigarette users

Overall, 12.3% of participants who used e-cigarettes bought them from supermarkets, 42.8% from small shops, 16.8% online and 55.3% acquired them from other sources (table 4). In the adjusted logistic regression, there was some evidence that sources of e-cigarettes changed over the survey years. In 2022, participants were twice as likely to source e-cigarettes from small shops (OR 2.02; 95% CI 1.24, 3.29) and 68% were less likely to source them online (OR 0.32; 95% CI 0.17, 0.58), compared with 2019. Younger e-cigarette users (16–17 and 14–15 years) were less likely to get their e-cigarettes from supermarkets and more likely to acquire them from other sources than 18 year-olds. Participants living in a household with someone who used e-cigarettes were more likely to source their e-cigarettes from supermarkets and small shops than those without an e-cigarette user in the household.

**Table 4:** Adjusted logistic regression for likelihood of acquiring e-cigarettes from different sources in UK adolescents (n=608)

	Supermarket		Small shop		Online		Acquired other	
Characteristic	n (%)	aOR(95% CI)	n (%)	aOR(95% CI)	n (%)	aOR (95% CI)	n (%)	aOR (95% CI)
Total sample	80 (12.3)		259 (42.82)		102 (16.8)		324 (55.3)	
Survey year								
2019	14 (9.3)	Ref	44 (34.2)	Ref	32 (25.6)	Ref	76 (56.7)	Ref
2020	16 (10.8)	1.15 (0.51, 2.58)	50 (39.9)	1.32 (0.75, 2.31)	16 (11.7)	0.36** (0.18, 0.76)	75 (61.4)	1.21 (0.70, 2.10)
2021	17 (15.4)	1.65 (0.70, 3.87)	39 (40.0)	1.31 (0.75, 2.32)	26 (22.9)	0.78 (0.41, 1.48)	58 (51.8)	0.83 (0.46, 1.48)
2022	33 (13.4)	1.31 (0.64, 2.70)	126 (51.2)	2.02** (1.24, 3.29)	28 (11.3)	0.32*** (0.17, 0.58)	115 (52.78)	0.88 (0.55, 1.43)
Gender								
Male	76 (56.3)	Ref	Ref	Ref	Ref	Ref	161 (52.9)	Ref
Female	75 (61.4)	0.97 (0.59, 1.60)	124 (41.6)	0.82 (0.58, 1.18)	45 (15.3)	0.87 (0.55, 1.40)	163 (58.3)	1.33 (0.93, 1.92)
Age								
18	40 (18.8)	Ref	91 (43.0)	Ref	35 (16.9)	Ref	86 (41.4)	Ref
16–17	27 (10.5)	0.54* (0.31, 0.95)	106 (40.0)	0.92 (0.63, 1.36)	44 (16.7)	1.02 (0.61, 1.71)	139 (56.5)	1.77** (1.20, 2.60)
14–15	7 (6.8)	0.33* (0.14, 0.77)	39 (41.1)	0.96 (0.58, 1.59)	16 (16.7)	1.04 (0.52, 2.08)	76 (74.9)	4.28*** (2.45, 7.47)
11–13	6 (12.6)	0.59 (0.23, 1.48)	23 (55.4)	1.82 (0.85, 3.91)	7 (17.3)	0.89 (0.37, 2.13)	23 (50.4)	1.44 (0.70, 2.98)
Social grade								
Middle class	60 (12.6)	–	190 (43.9)	–	79 (17.9)	–	241 (55.6)	–
Working class	20 (11.6)	0.95 (0.53, 1.70)	69 (39.8)	0.93 (0.62, 1.41)	23 (13.8)	0.74 (0.41, 1.31)	83 (54.4)	0.86 (0.57, 1.32)
Country								
England	70 (12.3)	–	235 (45.3)	–	92 (17.6)	–	268 (53.8)	–
Wales	4 (13.7)	1.05 (0.32, 3.41)	5 (22.1)	0.29* (0.10, 0.87)	5 (16.8)	0.89 (0.32, 2.51)	15 (57.2)	1.37 (0.57, 3.32)
Scotland	6 (11.7)	1.06 (0.42, 2.65)	19 (30.2)	0.50* (0.26, 0.94)	5 (8.5)	0.49 (0.18, 1.33)	41 (69.2)	1.95* (1.06, 3.59)
E-cigarette use in the household								
No	19 (6.7)	–	86 (34.3)	–	26 (10.2)	–	163 (63.9)	–
Yes	61 (16.4)	2.58** (1.47, 4.54)	173 (49.2)	1.69** (1.18, 2.43)	76 (21.6)	2.64*** (1.58, 4.41)	161 (48.9)	0.56** (0.39, 0.82)

N (%)=number and per cent of e-cigarette users who obtained cigarettes through this source within each stratum of the sociodemographic variable. E-cigarette sources are not mutually exclusive, therefore percentages do not add to 100% across sources.

*P<0.05, **p<0.01, ***p<0.001.

aORadjusted OR

Interactions between age and survey year were tested for all regression models and were found to be significant for buying e-cigarettes from small shops (figure 1B). Among 11 year-olds, there was a decreasing trend in the odds of buying cigarettes from small shops between 2018 and 2022, but the CIs overlapped. There was an increase in likelihood for 18 year-olds buying e-cigarettes from small shops, from around 20% in 2019 to 50% in 2022.

### Sensitivity analysis

To test if the results were robust to possible misclassification bias, the variables for noticing e-cigarettes and tobacco cigarettes in supermarkets and small shops were recoded, whereby not noticing additionally included the ‘hardly ever’ response. The proportion of participants who noticed both e-cigarettes and tobacco cigarettes was lower in the sensitivity analysis (online supplemental table 2). For example, in the main analysis, 64% of participants noticed cigarettes in supermarkets while 31% noticed them in the sensitivity analysis. Despite this change, the ORs in the main and sensitivity analyses were similar and the overall conclusions remained. For example, the lower likelihood of noticing tobacco cigarettes in supermarkets and small shops in 2022 compared with 2018 remained, as did the higher likelihood of noticing e-cigarettes on sale in supermarkets over time.

## Discussion

In this study of adolescents in Great Britain, we found that the likelihood of seeing e-cigarettes on display in shops increased over time while the likelihood of seeing tobacco cigarettes on display decreased. There was not strong evidence that sources of tobacco cigarettes changed over time, but there was a higher likelihood that e-cigarette users obtained their e-cigarettes from small shops in 2022 compared with 2019. Younger adolescents were more likely to acquire their nicotine products from other sources (eg, buying or being given by friends or family) and less likely to get them from supermarkets than those aged 18 years.

We found evidence that noticing tobacco cigarettes on sale fell over time but noticing e-cigarettes on display rose over time. This finding reflects in part the current policy landscape in the UK whereby only tobacco cigarettes are covered by point-of-sale display bans. The reason for these changes may include the rapid rise of disposable e-cigarette use among young people in the UK, driven in part by their low costs and advertising.^18^ Recent years have also witnessed an increase in involvement of the traditional tobacco industry in the e-cigarette market,^21^ and an increase in marketing, in particular online and on social media.^22^ Our study adds to other evidence including a 2022 cross-sectional study of e-cigarette displays and tobacco paraphernalia in two English cities. While this used a more detailed assessment method across a smaller area it found that display of these products was near-ubiquitous in shops visited.^23^ Exposure to e-cigarette displays has been cross-sectionally associated to e-cigarette use in Scottish children and adolescents^24^; therefore, a rise in noticing e-cigarette displays could impact e-cigarette uptake. However, e-cigarette use also rose during this time^18^; therefore, it is difficult to determine whether this is due to reverse causation, or if there is no relationship between these two.

Nicotine product users aged 14–17 years were significantly less likely to buy their nicotine product in supermarkets compared with users aged 18 years. Furthermore, it appears that the younger the user, the less likely they were to source their tobacco cigarettes from supermarkets over time, compared with older cigarette users. This suggests that minimum age restrictions on purchasing tobacco did affect direct purchases from supermarkets, in line with previous findings.^11^ Nonetheless, the fact that sources of tobacco cigarettes show no difference over time suggests that age of sale laws are currently underenforced, and supports calls for the UK to raise this to 21 years. Additionally, introducing and enforcing ‘Challenge 25’ measures to verify the age of potential customers would further address the consistent issue of availability of tobacco to children and adolescents. For purchases of e-cigarettes from small shops there was no difference by age. This finding is consistent with the literature which has also shown underage users are able to purchase e-cigarettes. For example, a study of the minimum age restriction policy for e-cigarettes in Scotland suggested that in the first year the likelihood of children purchasing e-cigarettes was not affected by the introduction of an age restriction.^25^

### Strengths and limitations

This study used a large representative sample of adolescents in Great Britain. The use of recent data (2018–2022) has permitted us to examine and account for the rising use of e-cigarettes in recent years, enhancing policy relevance. The data set had detailed data on nicotine product use enabling us to conduct a detailed analysis on noticing and acquiring these products, for the first time comparing the sources between countries, across years and by age.

There are some limitations. The levels of exposure to tobacco and e-cigarette displays reported in our main analyses are higher than in some other work, such as a study using survey data from 2011 to 2016 which found 28% of 11–16 year-olds reported seeing cigarette packets on display in 2016.^26^ It may be that the question wording used in our study for display of these products means that some participants include noticing products when they are being given to customers for example. We classified participants reporting that they ‘hardly ever’ saw products on display as noticing them in our main analysis. However, in sensitivity analyses we only included those responding that they saw these products as least ‘sometimes’. These analyses give similar trends and patterns, although with lower levels of exposure. It is also worth noting that our main aim was to assess changes in exposure over time, rather than levels of exposure per se.

Additionally, there was some missingness for questions regarding the display of nicotine products, consequently, the study lost power and the representativeness of the analysis may have been affected. Furthermore, due to low levels of tobacco and e-cigarette use, the sample sizes for some of the nicotine product sources were low, but comparable to other nicotine product use surveys.^19^ We additionally used slightly different definitions for tobacco cigarette and e-cigarette use. Participants were able to state multiple sources for their nicotine products, the binary variables used were not mutually exclusive. Therefore, this study cannot comment on the relationship between different sources. Participants also used their own judgement in determining what was a small shop and what was a supermarket, which may have introduced some misclassification. The data set was limited in detailed collection of sociodemographic data and as such has no information on ethnicity. We did identify some differences in product sources between the three countries in Great Britain, which is perhaps surprising as they have similar regulations. It should be noted, however, that sample sizes of product users in Scotland and Wales were low, and these differences should be interpreted with caution. Finally, it must be recognised that the COVID-19 pandemic might have affected the results. The 2020 data were collected in March 2020 and the 2021 data in March to April 2021. During this time, access to small shops and supermarkets was not restricted; however, adolescents’ social lives may well have been impacted by the pandemic.

## Conclusions

This study suggests that measures to limit access to tobacco and e-cigarettes, such as age restrictions, are not being adequately enforced. Policymakers need to be aware that additional policy approaches and enforcement will be required to effectively restrict awareness of and access to nicotine products for youth to blunt the trend for increased use in the age group.

## supplementary material

10.1136/tc-2023-058011 online supplemental file 1

## Data Availability

Data may be obtained from a third party and are not publicly available.
