# Supplementary material for: Changing awareness and sources of tobacco and e-cigarettes among children and adolescents in Great Britain
Source: Tob Control. 2023 Jul 30;33(e2):e058011. doi: 10.1136/tc-2023-058011 (PMC11672015; doi:10.1136/tc-2023-058011)
Supplement: online supplemental file 1 [file tc-33-e2-s001.pdf]

SUPPLEMENTARY FILES

Supplementary Table 1 – Categorisation of responses to questions on sources of e-cigarettes and tobacco cigarettes

<table>
<tr><th>Four binary variables</th><th>Multi-categorical options</th><th>Tobacco cigarette – open ended responses</th><th>E-cigarette – open ended responses</th></tr>
<tr><td>Bought: supermarket</td><td>I buy them from a supermarket</td><td></td><td></td></tr>
<tr><td rowspan="2">Bought: small shop</td><td>I buy them from a newsagent, tobacconist, or a sweet shop</td><td></td><td rowspan="2">“Vape shops”
“Vape shops”
“Vape store”
“Vape shop” (3)</td></tr>
<tr><td>I buy them from a petrol station or garage shop</td><td></td></tr>
<tr><td>Bought: online (e-cigs only)</td><td>I buy them through the Internet</td><td>N/A</td><td>“online” (2)
“On-line”
“Online from a vape store”
“Amazon”</td></tr>
<tr><td rowspan="11">Acquired other</td><td>I buy them from a machine</td><td rowspan="6">“Buy them abroad since they have been over 7 pound and got rid go 10 packs. As a light smoker It makes more sense as I never finish a pack”
“My friends go and buy them for me (those over 18)”
“Get big sister to go to shop for me”
“Older friends buy them for me”</td><td rowspan="6">“Someone else buys them from vape shop in town”</td></tr>
<tr><td>I buy them from some other type of shop</td></tr>
<tr><td>I buy them from street markets</td></tr>
<tr><td>I buy them from friends or relatives</td></tr>
<tr><td>I buy them from someone at school (not including friends)</td></tr>
<tr><td>I buy them from someone else</td></tr>
<tr><td>Friends give them to me</td><td rowspan="4">“Share with flatmates”
“Friends and strangers in smoking areas”</td><td rowspan="4">“I borrow my friends occasionally for a couple of puffs”
“I just use my friend's, who gets them from friends - both buying and being given them”
“Just lying round the house”
“Borrow from a friend”
“I only tend to smoke at parties”</td></tr>
<tr><td>My brother or sister gives them to me</td></tr>
<tr><td>My mother or father gives them to me</td></tr>
<tr><td>Someone else gives them to me</td></tr>
<tr><td>I take them</td><td>“Serbia”
“Rather illegally”
“I find them”
“I steal them from others”</td><td>“I don't buy them”</td></tr>
</table>

Supplementary Figure 1 – Flow diagram of sample exclusions in the analyses.

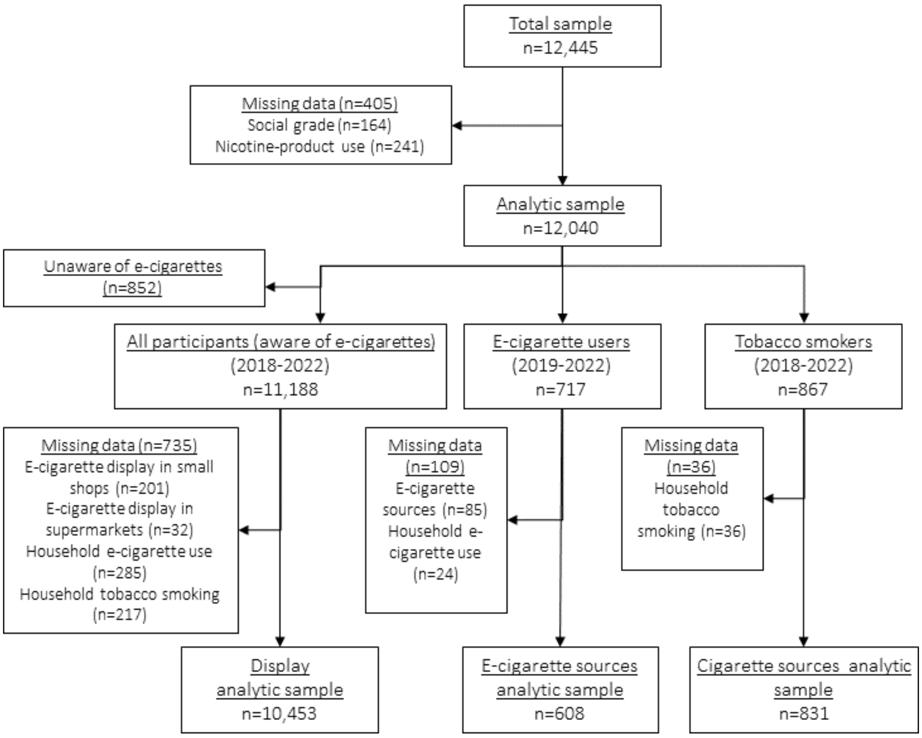

* All participants refers to all the participants who stated they were aware of e-cigarettes.

Supplementary Table 2 – Adjusted logistic regression showing the likelihood of noticing e-cigarettes and tobaccocigarettes on sale in supermarkets and small shops (n=10,453), with noticing recoded to include ‘every time’, ‘most times’ and ‘sometimes’ compared against ‘hardly ever’ and ‘never’

	Model 1: Tobacco cigarettes				Model 2: E-cigarettes			
	Supermarket		Small shop		Supermarket		Small shop	
Characteristic	N (%)	aOR (95% CI)	N (%)	aOR (95% CI)	N (%)	aOR (95% CI)	N (%)	aOR (95% CI)
Total Sample	3453 (31.05)		5453 (49.55)		3683 (33.76)		5100 (46.86)	
Survey year								
2018	583 (32.97)	*Ref.*	1025 (56.96)	*Ref.*	422 (23.73)	*Ref.*	779 (43.61)	*Ref.*
2019	803 (33.35)	1.04 (0.89, 1.21)	1175 (49.44)	0.73*** (0.63, 0.84)	808 (34.46)	1.78*** 1.53, 2.08)	1025 (43.84)	1.02 (0.89, 1.17)
2020	786 (33.17)	1.04 (0.90, 1.20)	1171 (49.97)	0.75*** (0.66, 0.87)	917 (39.57)	2.26*** 1.94, 2.63)	1157 (50.26)	1.35*** (1.18, 1.55)
2021	658 (28.5)	0.82** (0.70, 0.95)	1021 (45.07)	0.61*** (0.53, 0.70)	729 (32.66)	1.67*** 1.43, 1.96)	978 (43.67)	1.04 (0.90, 1.19)
2022	623 (27.38)	0.78** (0.67, 0.91)	1061 (47.57)	0.70*** (0.61, 0.81)	807 (36.36)	1.91*** 1.63, 2.23)	1161 (52.28)	1.46*** (1.27, 1.68)
Gender								
Male	1573 (29.46)	*Ref.*	2567 (48.21)	*Ref.*	1746 (33.05)	*Ref.*	2454 (46.32)	*Ref.*
Female	1880 (32.7)	1.21*** (1.10, 1.32)	2886 (50.94)	1.15** (1.05, 1.25)	1937 (34.5)	1.08 0.99, 1.19)	2646 (47.42)	1.06 (0.98, 1.16)
Age								
18	915 (46.45)	*Ref.*	1364 (69)	*Ref.*	849 (43.5)	*Ref.*	1156 (58.76)	*Ref.*
16-17	1342 (47.26)	1.07 (0.94, 1.21)	1918 (67.49)	0.96 (0.85, 1.09)	1272 (43.92)	1.10 0.97, 1.25)	1699 (58.86)	1.07 (0.94, 1.21)
14-15	561 (22.14)	0.35*** (0.30, 0.40)	1045 (40.75)	0.33*** (0.29, 0.37)	789 (30.74)	0.64*** 0.56, 0.73)	1128 (43.87)	0.60*** (0.53, 0.68)
11-13	635 (21.35)	0.34*** (0.29, 0.38)	1126 (37.29)	0.29*** (0.25, 0.33)	773 (25.95)	0.52*** 0.45, 0.59)	1117 (37.08)	0.46*** (0.41, 0.53)
Social grade*								
ABC1 (higher)	2545 (32.36)	*Ref.*	3973 (50.93)	*Ref.*	2591 (33.62)	*Ref.*	3624 (46.78)	*Ref.*
C2DE (lower)	908 (27.9)	0.80*** (0.72, 0.89)	1480 (46.25)	0.85*** (0.77, 0.93)	1092 (34.09)	1.00 0.91, 1.11)	1476 (47.04)	1.01 (0.91, 1.11)
Country								
England	3029 (31.49)	*Ref.*	4806 (50.59)	*Ref.*	3192 (33.93)	*Ref.*	4431 (47.17)	*Ref.*
Wales	151 (29.55)	0.92 (0.75, 1.14)	239 (46.17)	0.97 (0.77, 1.21)	173 (33.23)	1.00 (0.81, 1.25)	248 (48.58)	1.09 (0.87, 1.38)
Scotland	273 (27.12)	0.69*** (0.60, 0.81)	408 (40.38)	0.65*** (0.56, 0.77)	318 (32.25)	0.77*** (0.66, 0.89)	421 (42.37)	0.71*** (0.61, 0.84)
Current nicotine-product use status								
Non-user	2924 (29.27)	*Ref.*	4702 (47.65)	*Ref.*	3008 (30.95)	*Ref.*	4277 (44.1)	*Ref.*
E-cigarette only	161 (48.33)	1.51** (1.16, 1.97)	230 (68.63)	1.65*** (1.25, 2.16)	219 (65.19)	2.63*** 2.03, 3.41)	265 (79.08)	3.06*** (2.29, 4.10)
Tobacco cigarettes only	189 (45.75)	1.17 (0.92, 1.47)	291 (69.56)	1.45** (1.13, 1.85)	225 (54.96)	2.12*** 1.69, 2.66)	280 (66.03)	1.82*** (1.45, 2.29)
Dual-user	179 (55.29)	1.76*** (1.35, 2.30)	230 (69.85)	1.57** (1.19, 2.07)	231 (70.5)	3.03*** 2.32, 3.95)	278 (83.14)	3.78*** (2.77, 5.17)

	Model 1: Tobacco cigarettes				Model 2: E-cigarettes			
	Supermarket		Small shop		Supermarket		Small shop	
Characteristic	N (%)	aOR (95% CI)	N (%)	aOR (95% CI)	N (%)	aOR (95% CI)	N (%)	aOR (95% CI)
E-cigarette use in the household								
No	2762 (29.84)	*Ref.*	4437 (48.83)	*Ref.*	2748 (30.45)	*Ref.*	3959 (44.11)	*Ref.*
Yes	691 (36.63)	2.12*** (1.80, 2.51)	1016 (52.9)	1.60*** (1.35, 1.90)	935 (49.12)	1.56*** 1.32, 1.83)	1141 (59.62)	1.47*** (1.24, 1.73)
Tobacco smoking in the household								
No	3042 (29.62)	*Ref.*	4928 (48.56)	*Ref.*	3262 (32.43)	*Ref.*	4582 (45.65)	*Ref.*
Yes	411 (47.7)	1.20** (1.05, 1.36)	525 (61.07)	1.08 (0.96, 1.22)	421 (49.31)	1.86*** 1.64, 2.10)	518 (60.92)	1.56*** (1.39, 1.76)

N (%) = Number and percent of participants who noticed e-cigarettes or tobacco cigarettes on display within each stratum of the sociodemographic variables; aOR = adjusted Odds Ratio; 95% CI = 95% confidence interval; *p<0.05; **p<0.01; ***p<0.001;

*Social grade (based on National Readership Survey (NRS) classification of occupations and classified as ABC1 (higher) vs. C2DE (lower)).
